# Diversity and function of soybean rhizosphere microbiome under nature farming

**DOI:** 10.3389/fmicb.2023.1130969

**Published:** 2023-03-01

**Authors:** Dominic V. A. Agyekum, Tatsuyuki Kobayashi, Khondoker M. G. Dastogeer, Michiko Yasuda, Elsie Sarkodee-Addo, Safirah T. N. Ratu, Qicong Xu, Takaaki Miki, Eri Matsuura, Shin Okazaki

**Affiliations:** ^1^United Graduate School of Agricultural Science, Tokyo University of Agriculture and Technology, Fuchu, Japan; ^2^Faculty of Agriculture, Tokyo University of Agriculture and Technology, Fuchu, Japan; ^3^Department of Plant Pathology, Faculty of Agriculture, Bangladesh Agricultural University, Mymensingh, Bangladesh; ^4^Institute of Global Innovation Research, Tokyo University of Agriculture and Technology, Fuchu, Japan; ^5^International Nature Farming Research Center, Nagano, Japan; ^6^College of Agriculture, Ibaraki University, Mito, Japan

**Keywords:** nature farming, microbiome, rhizosphere, symbiosis, fumigation, soybean

## Abstract

Nature farming is a farming system that entails cultivating crops without using chemical fertilizers and pesticides. The present study investigated the bacterial and fungal communities in the rhizosphere of soybean grown in conventional and nature farming soils using wild-type and non-nodulating mutant soybean. The effect of soil fumigant was also analyzed to reveal its perturbation of microbial communities and subsequent effects on the growth of soybean. Overall, the wild-type soybean exhibited a better growth index compared to mutant soybean and especially in nature farming. Nodulation and arbuscular mycorrhiza (AM) fungi colonization were higher in plants under nature farming than in conventionally managed soil; however, fumigation drastically affected these symbioses with greater impacts on plants in nature farming soil. The rhizosphere microbiome diversity in nature farming was higher than that in conventional farming for both cultivars. However, the diversity was significantly decreased after fumigation treatment with a greater impact on nature farming. Principal coordinate analysis revealed that nature farming and conventional farming soil harbored distinct microbial communities and that soil fumigation significantly altered the communities in nature farming soils but not in conventional farming soils. Intriguingly, some beneficial microbial taxa related to plant growth and health, including *Rhizobium*, *Streptomyces*, and *Burkholderia*, were found as distinct microbes in the nature farming soil but were selectively bleached by fumigant treatment. Network analysis revealed a highly complex microbial network with high taxa connectivity observed under nature farming soil than in conventional soil; however, fumigation strongly broke it. Overall, the results highlighted that nature farming embraced higher microbial diversity and the abundance of beneficial soil microbes with a complex and interconnected network structure, and also demonstrated the underlying resilience of the microbial community to environmental perturbations, which is critical under nature farming where chemical fertilizers and pesticides are not applied.

## 1. Introduction

Agriculture is critical to meeting the food needs of the ever-growing human population. Since the green revolution, most agricultural management practices have relied on the use of chemical fertilizers and pesticides to increase crop production. The use of these agrochemicals has led not only to increased crop yields but also to several environmental problems, including soil degradation, leaching of agrochemicals into surface water bodies, the emergence of pesticide-resistant species, and loss of soil microbial biodiversity, resulting in an unsustainable agricultural practice (Singh and Mondal, 2017; Kumar et al., 2021). Consequently, current agricultural practices must be considerably modified to reduce the use of excessive chemicals and their impact on the environment, while still maintaining high crop yields.

The goal of sustainable agriculture is to maintain the biological function of the soil and to promote plant health. “Nature farming,” which originated in Japan, contributes to this goal by using techniques such as crop rotation and green manuring to maintain soil fertility and crop growth, instead of using chemical fertilizers and pesticides. Nature farming differs from other organic farming systems in that it strictly prohibits the use of untreated animal manure and urban sewage because improperly composted sewage and manure (Martin, 2005) can be a source of pathogen and heavy metal contamination for crop products meant for human consumption (Xu, 2001). As a result, when compared to conventional farming, nature farming is more sustainable, cost-effective, environmentally friendly, and improves soil fertility and biodiversity.

Under nature farming systems, plant health and productivity are largely influenced by root microbiota. Plants benefit from root-associated microbes in various ways, including nutrient acquisition, pathogen resistance, and stress tolerance (Bulgarelli et al., 2013). The rhizosphere is the immediate narrow zone surrounding plant roots and is inhabited by an immense population of microbes (Hartmann et al., 2008). Several root-associated bacteria and fungi have been identified as plant-growth-promoting microorganisms that play a variety of essential roles in regulating the growth and yield of plants. Rhizobia are the most well-known mutualistic bacterial symbionts, capable of fixing atmospheric nitrogen in the roots of leguminous plants through nodulation (Sugiyama, 2019). Likewise, arbuscular mycorrhizal fungi (AMF) are the most common mutualistic fungal symbionts that establish symbiosis with a majority of land plants (Smith et al., 2011) and benefiting host plants in a variety of ways, including the supply of inorganic phosphorus and improved stress and disease tolerance in plants (Berendsen et al., 2012; Haney et al., 2015; Li et al., 2021). Recently, some studies have evaluated the impact of fertilizer application on microbial community structure under conventional and nature farming systems (Liao et al., 2019; Sinong et al., 2021). These studies indicated a higher microbial abundance and diversity in nature farming soils than in conventional farming soils (Liao et al., 2019; Sinong et al., 2021). Regardless of these fundamental works, little is known about the composition and function of the root-associated microbial communities associated with plants under nature farming systems, requiring further research on the functions of microbial communities in nature farming soils. For instance, no study has explored the rhizosphere microbiome of nature farming soybean in Japan despite soybean being one of the most cultivated crops under nature farming system.

Soil fumigation has been extensively used to control soil-borne pathogens and weeds in various farming systems (Strauss and Kluepfel, 2015). Chloropicrin has been commonly used as soil fumigant after methyl bromide was phased out in many developed countries (Mao et al., 2014). Even though chloropicrin has shown promising biocidal activity, such fumigants are non-selective biocides with deleterious effects on non-target organisms, including beneficial soil microorganisms. Very little is known about the effects of fumigants on legume production, especially on the symbiotic association of legumes with rhizobia, mycorrhizal fungi, and other beneficial soil symbionts. Evaluating the impact of soil fumigants on symbiotic systems will provide essential fundamental information for the long-term and effective use of beneficial soil microorganisms in agricultural production.

The present study tested the hypothesis that nature farming harbors a distinct and diverse rhizosphere microbiome that promotes plant growth using soybean as the model system. The effect of soil fumigant on the rhizosphere microbiome of soybean was also investigated. With the use of high-throughput sequencing of 16S rRNA and ITS gene markers, the bacterial and fungal rhizosphere microbiome of soybean under nature farming conditions was assessed and compared to that of conventional farming systems.

## 2. Materials and methods

### 2.1. Soil sampling and fumigation by chloropicrin

Nature farming soil was collected on 5th June 2021 from the International Nature Farm Research Center experimental field (Nagano, Japan, 36°11′57.5″N 137°52′27.7″E), which has continuously practiced no chemical fertilizer and pesticide application for more than 10 years. Conventional farming soil was collected on 6th June 2021 from the Tokyo University of Agriculture and Technology experimental field (Tokyo, Japan, 35°41′02.3″N 139°29′03.9″E). Both soils are classified as Andosols according to the Japanese soil classification system (Amano, 1985). The soil samples were collected from the top 15 cm of soil. Sub-samples were air dried and passed through a 2 mm sieve to obtain fine earth fractions for routine soil physicochemical analyses. Each of the field soil samples was divided into two parts: fumigant treatment and non-fumigated soil samples. Chloropicrin (Mitsui Chemicals Agro Co., Ltd., Tokyo, Japan) was used as the fumigant and applied as an aqueous solution at the recommended rate of 12 ml per 6 kg of soil. The soil was sealed in a plastic bag and mixed every 2 days for a period of 10 days and then opened for aeration for 2 days. Approximately 2.5 L of soil was used to fill 1/5,000 Wagner pots for soybean cultivation.

### 2.2. Soil chemical analysis

Soil pH and electrical conductivity (EC) were measured in 1 M KCl and deionized water at a soil-to-solution ratio of 1:5. Total carbon (TC) and total nitrogen (TN) were measured using the dry combustion method and an NC analyzer (SUMIGRAPH NC TR22, Sumika Chemical Analysis Service Ltd., Osaka, Japan). Inorganic N in the soil was extracted from soil using 2 M KCl and N-NH_4_^+^ in the extract was analyzed by the modified indophenol blue method, and N-NO3-in the extract was analyzed by flow injection analysis using a flow-through visible spectrophotometer equipped with a copperized cadmium column to reduce nitrate to nitrite in the sample solutions. Available phosphate (Available-P) was evaluated by the Bray 2 method.

### 2.3. Soybean cultivation

Two soybean cultivars (Glycine max L.), Enrei (wild-type), and its non-nodulating mutant, En1282 were used in this experiment. All soybean seeds were surface sterilized by immersion in 20 ml caplate (Mitsui Chemicals Agro Co., Ltd., Tokyo, Japan) solution (10 mg/mL) for 1 h at 28°C. The seeds were then washed with sterilized distilled water several times, placed on sterilized moist tissue paper, and incubated for 72 h at 25°C under dark conditions for germination. The germinated seeds were sown directly into the prepared pots, with two seeds per pot and 12 replications. The plants were grown in a phytotron (20–25°C day/night) and watered regularly. SPAD value and stem length were measured weekly from 3 weeks after planting until the tenth week. A copper-based fungicide, Bordo mix (Nihon Nohyaku Co., Ltd., Japan) was diluted 1:500 times and sprayed on the leaves of soybean cultivated in the fumigated soils 3 weeks after planting.

### 2.4. Rhizosphere soil collection

At 10 weeks after sowing, four plants from each treatment were harvested for rhizosphere microbiome analysis, while the remaining plants were grown for 12 weeks. The soybean plants were gently removed from the pots, and soil loosely attached to the roots was removed by mild shaking. The nodules were detached from the roots and counted, and the fresh weight of the roots was also measured. The soybean rhizosphere soil was collected according to the modified procedure of McPherson et al. (2018). Briefly, 2 g of the roots with tightly bound soil was put into a 50-ml centrifuge tube filled with 30 ml of autoclaved phosphate buffer (g/L: 6.33 g NaH_2_PO_4_.H_2_O, 16.5 g Na_2_HPO_4_.7H_2_O, 200 μl surfactant, pH 6.5). The tube was vortexed at maximum speed (rpm) for 2 min, and the slurry was filtered through a 100-μm-cell strainer into a new 50-mL tube. The slurry was then centrifuged at 3,000 × *g* for 5 min to precipitate the soil particles. The pellet was resuspended in 1.5 ml phosphate buffer (without surfactant) and then pipetted into a 2 ml microfuge tube. The tube was centrifuged at 15,871 × *g* for 2 min, after which the supernatant was poured off and the soil pellet stored at −20°C until further processing.

### 2.5. Evaluation of AM fungi colonization

To determine the extent of AM colonization, the roots used to collect the rhizosphere soil were placed in 50-mL tubes. The roots were cleared with 10% KOH in a water bath for 15 min, acidified with 5% HCl, and stained with 0.05% trypan blue in lactoglycerol (Phillips and Hayman, 1970). Ten root fragments (1 cm long) per treatment were mounted on glass slides in three replicates to assess the morphological structures of AMF in the roots, which included hyphae, vesicles, and arbuscules following the procedure of McGonigle et al. (1990).

### 2.6. Soil DNA extraction and amplicon analysis

Rhizosphere soil DNA was extracted using the Nucleospin soil kit (MACHEREY-NAGEL, Dueren, Germany) following the manufacturer’s protocol. Amplicon sequencing of the bacterial V3/V4 region and fungal ITS region was carried out using the Miseq platform at Bioengineering Lab. Co. (Sagamihara, Japan). The bacterial V4 region was amplified using the forward primer 1st_PCR_515F_MIX:(5′-ACACTCTTTCCCTACACGACGCTCTTCCGATCT-NNNNN-GTGCCAGCMGCCGCGGTAA-3′) and the reverse primer 1st_PCR_806R_MIX:(5′-GTGACTGGAGTTCAGACGTGTGCTCTTCCGATCT-NNNNN-GGACTACHVGGGTWTCTAAT-3′), and the internal transcribed spacer (ITS) 1 region for fungi was amplified using the forward primer ITS1F_KYO1 (5′ACACTCTTTCCCTACACGACGCTCTTCCGATCTCTHGGTCATTTAGAGGAASTAA3′) and the reverse primer ITS2_KYO2 (5′GTGACTGGAGTTCAGACGTGTGCTCTTCCGATCTTTRCTRCGTTCTTCATC-3′) by PCR. The PCR reaction containing the DNA template (5 ng) was carried out in a final volume of 20 μl using the ExTaq polymerase kit (Takara). The PCR products were purified using AMPure XP (Beckman coulter) and quantified using Synergy H1 (Bio Tek) and QuantiFlour dsDNA system. A quality check on the libraries was done using the Fragment Analyzer and sdDNA 915 Reagent Kit (Advanced Analytical Technologies). The libraries were pooled together and loaded into the Illumina MiSeq instrument following the manufacturer’s instructions (Illumina, San Diego, CA, United States).

### 2.7. Bioinformatics

The Quantitative Insights into Microbial Ecology (Qiime 2.0) toolkit was used to process the raw high-throughput sequencing data. Then, the data obtained from the 16S, and ITS amplicon were analyzed using MicrobiomeAnalyst (Dhariwal et al., 2017), and the OTUs were annotated as SILVA labels.

### 2.8. Sequencing data processing and identification of amplicon sequence variants

Microbiome bioinformatics and identification were carried out with QIIME2 2020.8 (Bolyen et al., 2019). The adapter and primers were removed with Cutadapt v2.4 from the raw reads (Martin, 2011). The sequences were demultiplexed using the q2-demux plugin followed by quality control, length trimming, denoising, chimera, and PhiX removal, and feature table construction by DADA2 with default settings except for “-ptrunc-len-f” and “-p-trunc-len-r,” which were set at 250 and 200, respectively, for 16S data and at 160, and 200, respectively, for ITS data (*via* q2-dada2; Callahan et al., 2016). The resulting ASVs were aligned with mafft (Katoh et al., 2002). Taxonomy was assigned to ASVs using qiime feature-classifier classify-sklearn (Bokulich et al., 2018) with the pre-trained naïve Bayes SILVA classifier v132 trimmed to the V4 region of the 16S rDNA gene (Quast et al., 2012) for bacteria and pretrained UNITE ver8 99% database (UNITE Community, 2019), trained on the full reference sequences without any extraction for fungi. Non-bacterial and fungal reads were removed from the obtained ASV table. We normalized the library using scaling with ranked subsampling using he “SRS”-function in the “SRS” with “qiime srs SRS” (Beule and Karlovsky, 2020). Differences in microbial community diversity were evaluated as Shannon diversity index in qiime2 and Kruskal-Wallis tests were used to compare the diversity. Box plots to display the alpha diversity indices were created using ggplot2 (Wickham et al., 2016) installed in R Core Team (2020). The Bray–Curtis dissimilarity matrix was measured for bacterial and fungal communities and used for PCoA ordination plot in PAST4.04 (Hammer et al., 2001). We compared differences in community composition and structure among samples (β-diversity) using analysis of similarity (ANOSIM; Clarke, 1993) and Permutational Multivariate ANOVA (PERMANOVA; Anderson, 2014). Least discriminant analysis was performed using microbiomeAnalyst (Chong et al., 2020). Bacterial functions were predicted by PICRUSt2 software (ver. 2.3.0 b) based on the KEGG functional database (Douglas et al., 2020).

### 2.9. Statistical analysis

ANOVA was used to determine where treatment means values differed significantly (*p* < 0.05) from one another, and the SNK test was used to compare sets of means. The computations were carried out using routines built into the Microsoft Excel software ystat2013.

## 3. Results

### 3.1. Soil chemical properties

Soil chemical analysis was performed on samples collected from conventional and nature farming fields before crop cultivation. Both soils were characteristically acidic, with nature farming soil recording a slightly lower pH than conventional farming soil (Supplementary Table 1). The total carbon and nitrogen contents and ammonium nitrogen (NH_4_^+^-N) levels were higher in conventional farming soil while the available P content and nitrate nitrogen (NO_3_^−^-N) levels were higher in nature farming soil (Supplementary Table 1).

### 3.2. Soybean growth performance

In terms of shoot length, no significant differences in mean values were observed between wild-type plants grown in conventional and nature farming soils, irrespective of fumigation treatment (Table 1). Similarly, irrespective of the farming system or fumigant treatment, the shoot lengths of the mutant plants showed no significant differences; however, they were all significantly lower when compared to the wild type (Table 1), indicating the contribution of nitrogen supply by nitrogen-fixing nodules. No significant differences were found in the fresh root weight of all treatments except for the mutants grown in the non-fumigated conventional soil (Table 1). In conventional farming soil, chloropicrin treatments did not change the growth and the yields of wild-type soybean significantly; however, in nature farming soil, chloropicrin treatments significantly decreased the number of seed and seed pods of wild-type soybean (Table 1), suggesting that fumigation had a greater impact on nitrogen-fixing rhizobia in nature farming than conventional farming soil. In conventional farming soil, chloropicrin treatments increased the number of seeds especially in non-nodulating mutant soybean (Table 1), most probably because microbial decomposition by chloropicrin increased the amount of available nitrogen. On the contrary, in nature farming soil, chloropicrin treatment significantly decreased the yield of non-nodulating mutant soybean (Table 1), suggesting that microbes other than rhizobia greatly contribute to the yield of soybean in this farming system.

**Table 1:** Growth characteristics of *Glycine max* cv. Enrei (wild-type) and En1282 (non-nodulating mutant) grown under fumigated and non-fumigated nature and conventional farming systems.

Treatment	Shoot length (cm)	Root length (cm)	Chlorophyll content (SPAD)	No. of seeds/plant	No. of seed pod/plant
CncWT	30.1 ± 1.5a	3.39 ± 1.37a	28.3 ± 3.4a	10.3 ± 2.9b	6.3 ± 1.5bc
CncMT	21.6 ± 0.3c	2.66 ± 1.28b	21.0 ± 3.8b	7.1 ± 2.3c	5.1 ± 2.0c
CchloWT	28.6 ± 2.4ab	5.19 ± 1.72a	34.1 ± 2.9a	13.6 ± 1.5ab	8.0 ± 1.1ab
CchloMT	22.6 ± 0.3c	4.59 ± 0.78a	31.9 ± 3.9a	11.9 ± 1.8b	7.0 ± 1.2ab
NncWT	31.5 ± 2.4a	4.73 ± 0.92a	32.4 ± 6.4a	15.8 ± 4.1a	8.1 ± 2.7ab
NncMT	24.5 ± 1.4c	5.95 ± 1.02a	22.6 ± 3.9b	15.6 ± 3.6a	9.4 ± 1.6a
NchloWT	29.6 ± 1.3a	5.10 ± 0.89a	32.5 ± 3.1a	5.9 ± 1.1c	4.3 ± 1.0 cd
NchloMT	22.8 ± 1.4c	4.81 ± 1.34a	29.5 ± 2.6a	4.4 ± 1.8c	3.1 ± 1.1d

Data are mean ± SD (*n* = 4). Different lower cases in the same column show significant differences between treatments (*p* < 0.05). “C” and “N” represent conventional and nature farming systems, respectively. Chlo, chloropicrin fumigant; nc, no-fumigant; WT, Enrei cv. (wild-type), and MT, En1282 (non-nodulating mutant).

### 3.3. Nodulation and AM fungi colonization

**Figure 1:**
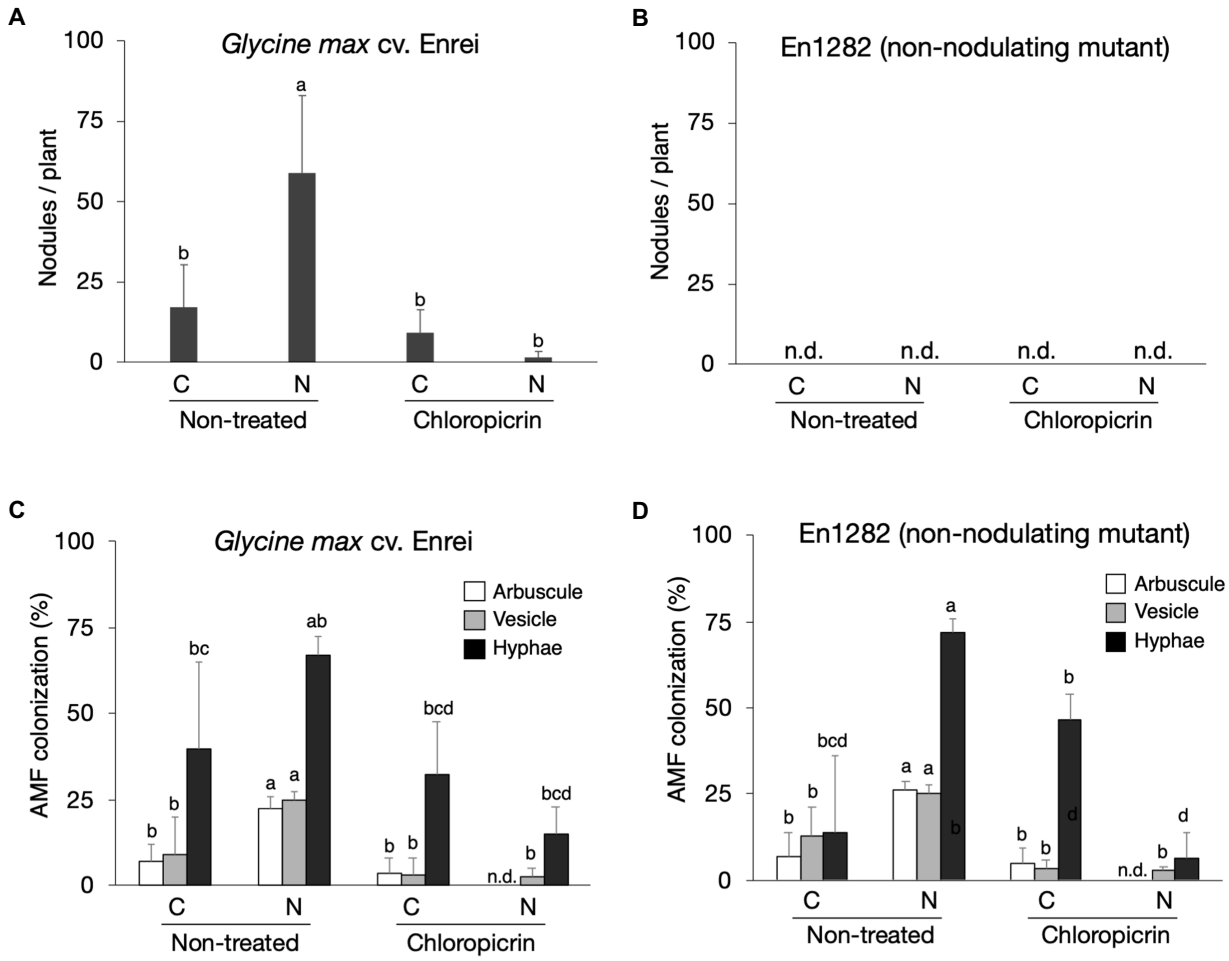
The effect of soils, fumigation, and soybean genotypes on average number of nodules **(A,B)** and the rate of AMF colonization in soybean roots based on the presence of arbuscles, vesicles, and hyphae **(C,D)**. Different letters indicate significant differences between samples based on the SNK test (*p* < 0.05). C, conventional farming soil, N, nature farming soil, *n* = 4.

Soybean cultivar, Enrei, and its non-nodulating mutant, En1282, were grown in conventional and nature farming soils, with and without fumigation treatment. As expected, no nodules were found on the roots of mutant (non-nodulating) plants (Figure 1B), whereas 17 and 59 nodules per plant on average were found on the roots of wild-type plants grown in conventional and nature farming soils, respectively, (Figure 1A). Fumigation however decreased the number of nodules under both farming systems, especially drastically in the nature farming soil (Figure 1A). There was a highly significant interaction (*p* < 0.001; Supplementary Table 2) between farming system, fumigation treatment, and soybean cultivar on the number of nodules formed.

Microscopic observations of the stained soybean roots showed the presence of AM fungi morphological structures including hyphae, vesicles, and arbuscles (Supplementary Figure 1). The arbuscule and vesicle percent colonization rates for both wild-type and mutant plants were typically noticeably higher when grown in nature farming than in the conventionally managed soil when no fumigant was applied (Figures 1C,D). The percent hyphae colonization was higher in naturally farmed soybean plants (66.8% for wild-type; 72.0% for mutant) than in conventionally farmed plants (40.0% for wild-type; 13.8% for mutant), though these differences were not statistically significant especially for the wild-type plants (Figures 1C,D; Supplementary Table 2). Fumigating the soils with chloropicrin resulted in a significant decrease in AM fungi root colonization rates of the soybean plants under both conventional and nature farming. It was determined that the percent AM fungi colonization in the soybean plants were not affected by an interaction effect. However, it was significantly affected by main effects of farming system (*p* < 0.001) and fumigation treatment (*p* < 0.001; Figures 1C,D; Supplementary Table 2). The average AM fungi colonization rates after fumigation treatment were higher in plants grown in conventional farming than in nature farming (Figures 1C,D) indicating that the fumigant applied had a severe impact on the plants in nature farming.

### 3.4. Amplicon sequencing results

High-throughput Illumina MiSeq amplicon sequencing produced 1,114,090 16S and 895,540 ITS reads, distributed across 24 samples belonging to 4,919 and 2,369 distinct microbial amplicon sequence variants (ASVs) for bacteria and fungi, respectively. Rarefaction curves increased steadily as the number of sequences increased (Supplementary Figure 1), indicating that the sample size was adequate for further analysis.

### 3.5. Soybean rhizosphere microbiome under nature farming is more diverse and distinct than in conventional farming

**Figure 2:**
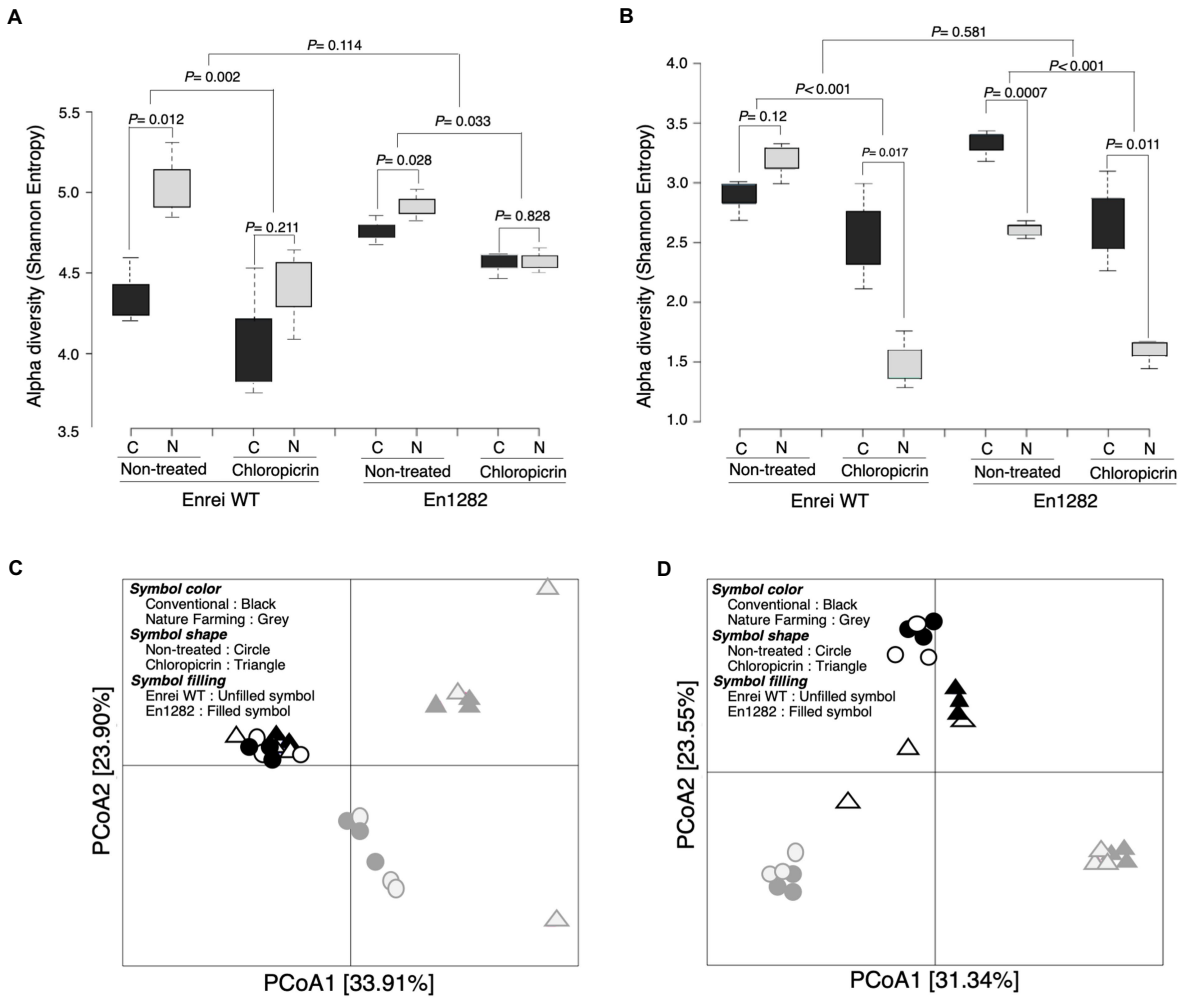
Rhizosphere bacterial and fungal community of soybean as influenced by cultivar, farming system, and chloropicrin treatment. Alpha diversity (Shannon index) of bacteria **(A)** and fungi **(B)**. Principal coordinate analysis (PCoA) based on Bray Curtis dissimilarity index for bacteria **(C)** and fungi **(D)**. C, conventional farming soil, N, nature farming soil.

Alpha diversity analyses based on the Shannon and Chao1 indexes revealed a more diverse and rich bacterial community in the rhizosphere of naturally managed soybean plants than in conventionally managed plants (Figure 2A; Supplementary Table 3). This effect was, however, more pronounced in the rhizosphere of wild-type soybean than in the mutant, even though the soybean cultivar did not significantly influence the rhizosphere alpha diversity (Figure 2A; Supplementary Table 3). A similar trend was observed in the fungal alpha diversity (both Shannon and Chao1) except for a significantly higher Shannon index observed in the rhizosphere of mutant plants grown in conventional than nature farming soil (Figure 2B; Supplementary Table 4).

According to the results of the differences between samples (β-diversity) using unconstrained principal coordinate analysis based on the Bray Curtis distance, there was a clear separation along axis 1 for bacteria (Figure 2C), and along axis 2 for fungi (Figure 2D), confirming that the conventional and nature farming soils harbored distinct microbiomes. At the same time, there was no clear separation of the wild-type or mutant plants grown in either of the farmed soils, suggesting a negligible effect of the cultivar on the community structure, which was further confirmed by PERMANOVA and ANOSIM tests (Supplementary Figure 3).

### 3.6. Diversity of soybean rhizosphere microbiome is affected in response to soil fumigation

The fumigant had a deleterious impact on the soybean rhizosphere microbial diversity, as evidenced by significantly lower alpha diversity in the rhizospheres of plants grown in fumigated soils compared to non-fumigated soils (Figures 2A,B). Following fumigation, the rhizosphere diversity indices (Shannon and Chao1) were similar between conventional and nature farming soil (Figure 2A; Supplementary Table 3). There was no discernible difference in alpha diversity between plants growing in fumigated conventional and nature farming soil for bacteria, but there was a significant difference in fungal alpha diversity, with the fumigant having a pronounced effect in the rhizosphere of plants growing in nature farming soil (Figures 2A,B). Interestingly, the bacterial species diversity of plants growing in the fumigated nature farm soil was higher than in the non-fumigated conventional farm soil (Figure 2A).

Principal coordinate analysis analysis of the Bray–Curtis distance illustrated significant differences in rhizosphere microbial community structure between the fumigated and non-fumigated treatments and this was reinforced by PERMANOVA and ANOSIM tests (Figures 2C,D; Supplementary Figure 3). The rhizosphere communities (bacteria and fungi) of plants growing in the fumigated and non-fumigated conventional farm soil were clustered together (Figures 2C,D), indicating the microbial communities were similar. However, those of plants grown in the nature farming soil were relatively distinct from each other (Figures 2C,D), indicating a stronger effect of the fumigants on the microbial communities in the nature farming soil.

### 3.7. The influence of farming system on soybean rhizosphere microbiome composition

**Figure 3:**
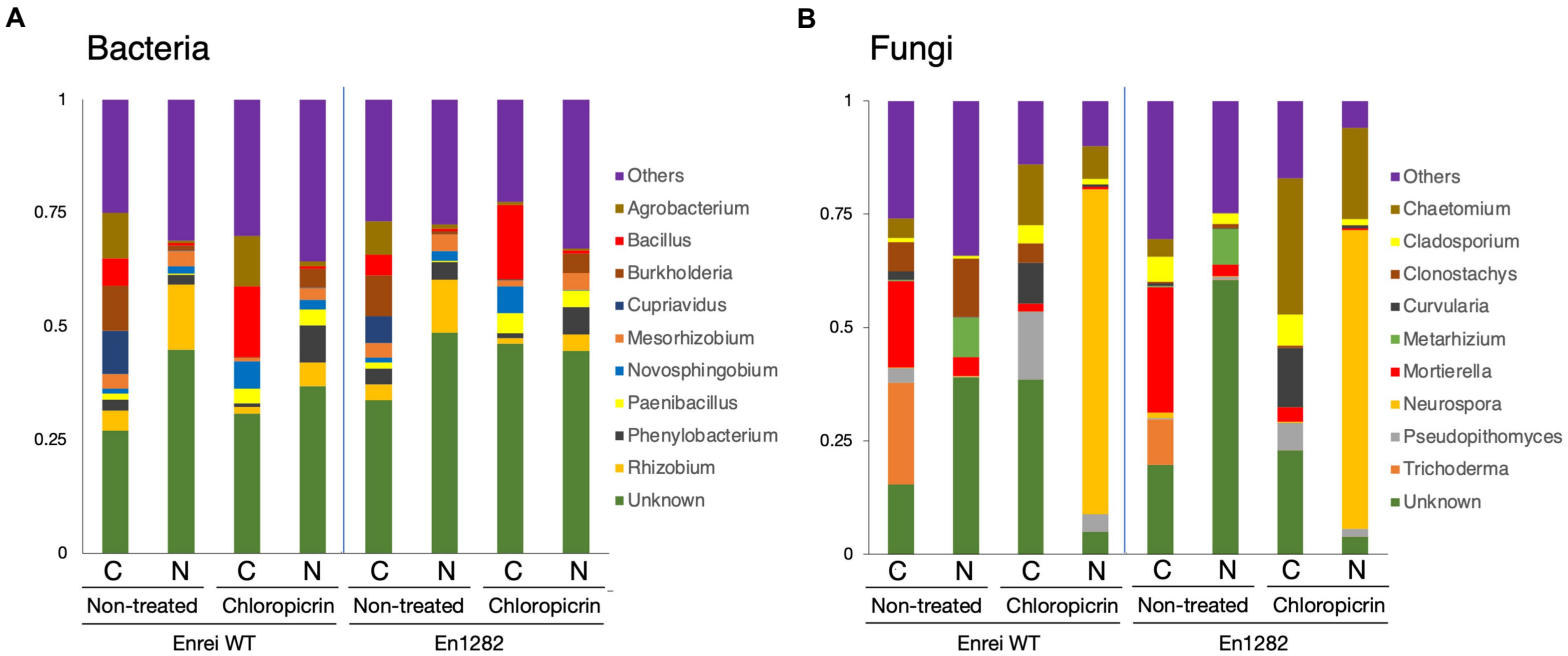
Taxonomic composition of microbial communities inhabiting the rhizosphere of soybean grown in conventional and nature farming soils with and without chloropicrin treatment. The bar graphs represent the relative abundance of bacterial **(A)** and fungal **(B)** communities at the genus level. Only taxa with abundance >1% are shown. WT = Enrei cv. C, conventional farming soil, N, nature farming soil.

The composition of bacterial and fungal communities was mostly different between the farming systems in terms of the abundance or absence of certain taxa. Proteobacteria was the most abundant bacterial phyla across all samples accounting for 34.5–65.8% of the total bacterial reads (Supplementary Figure 4A). Firmicutes were more abundant in the conventional farming soil while Acidobacteria and Bacteroidetes were dominant in the nature farming soil (Supplementary Figure 4A). The top 10 bacterial genera are shown in Figure 3A, of which the majority of them belonged to Proteobacteria. *Agrobacterium*, *Bacillus*, and *Cupriavidus* tended to be more abundant in the rhizosphere of plants grown in conventional farming soil while *Rhizobium* was more dominant in plants growing in nature farming soil. Unclassified bacteria accounted for ~30.0–48.6% in relative abundance across all rhizosphere samples.

With respect to fungal taxonomic composition, Ascomycota was the dominant phylum ranging from 37.1 to 97.2% across all treatments (Supplementary Figure 4B). Other often-reported fungal phyla, such as Basidiomycota, Chytridiomycota, and Mortierellomycota, were found in very low abundance, and their distribution varied across treatments (Supplementary Figure 4B). *Mortierella* and *Trichoderma* dominated the rhizosphere of soybean plants in the conventional farming soil while *Clonostachys* and *Metarhizium* were more dominant in the nature farming soil (Figure 3B).

### 3.8. Soil fumigation affects the rhizosphere bacterial and fungal community composition of soybean

Fumigation led to a change in the relative abundances of certain microbial taxa. The representation of Firmicutes and Bacteroidetes sharply increased, noticeably in the conventional and nature farming soil respectively, while the abundance of Proteobacteria relatively decreased under both farming systems (Supplementary Figure 4A). Chloroflexi and Acidobacteria were depleted or eliminated after fumigation and the effect was more pronounced in the nature farming soil (Supplementary Figure 4A). Fumigation resulted in the abundances of *Bacillus*, *Novosphigobium*, and *Paenibacillus* specifically increased in the rhizosphere of plants in the conventional farming soil, while *Phenylobacterium* was more in the rhizosphere of plants in the nature farming soil (Figure 3A). *Burkholderia*, *Cuprividus*, *Rhizobium*, and *Mesorhizobium* all decreased in relative abundance under both farming systems after fumigation (Figure 3A).

Regarding the fungal composition, fumigation led to an increase in the proportion of Ascomycota while the abundance of Mortierellomycota, Chytridiomycota, and Basidiomycota was decreased (Supplementary Figure 4B). The fungal genera were differentially represented among the treatments. For example, *Chaetomium* and *Neurospora*, which were not detected in the rhizosphere of plants grown in the nature farming soil, suddenly emerged, and responded strongly with higher abundance after fumigation (Figure 3B). There was a marked increase in the abundance of *Curvularia* and *Pseudopithomyces* with a higher distribution in the conventional than in nature farming soil (Figure 3B). On the contrary, *Trichoderma* and *Metarhizium*, detected only in the conventional and nature farming soil, respectively, were eliminated after fumigation (Figure 3B). Similarly, the abundance of *Mortierella* decreased significantly after fumigation and was more pronounced in the rhizosphere of plants grown in conventional farming soil (Figure 3B).

### 3.9. Differentially abundant taxa influenced by farming system and soil fumigation

**Figure 4:**
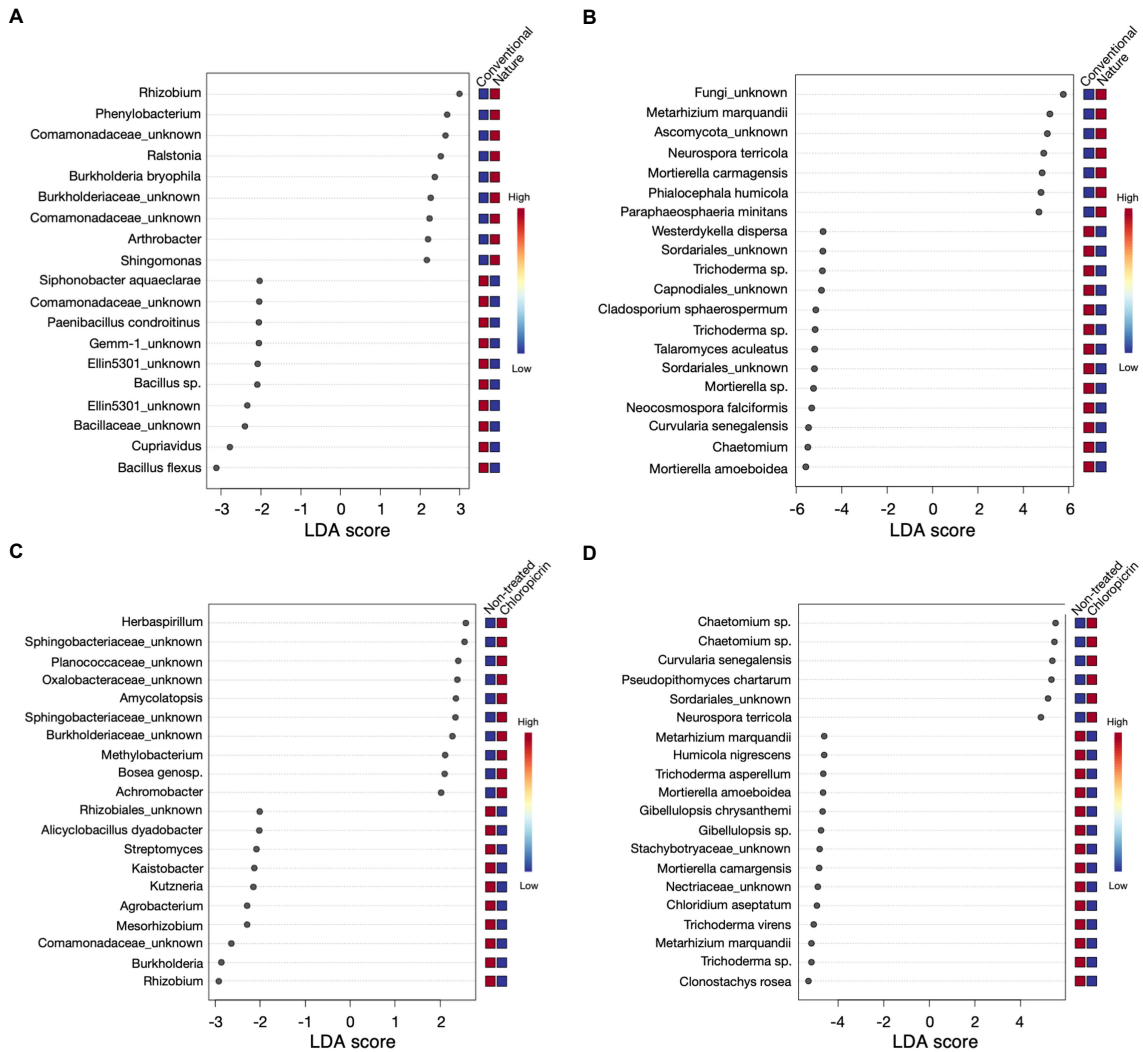
Linear discriminant analysis effect size (LEfSe) results of top 20 bacteria **(A)** and fungi **(B)** that were significantly enriched in either conventional or nature farming soil, and bacteria **(C)** and fungi **(D)** with significant abundance in either non-treated or chloropicrin-treated soils. Red boxes show a high abundance and blue boxes show a low abundance of a particular genera.

Linear discriminant effect size analysis (LefSe) was conducted to identify differentially abundant taxa that were significantly influenced by the farming system or fumigation treatment at an LDA score threshold of two. For the bacterial taxa, ASVs classified as *Rhizobium*, *Phenylobacterium*, and some members of the family *Comamonadaceae*, among others, were significantly enriched in the rhizosphere of plants grown in the nature farming soil (Figure 4A). In contrast, *Bacillus* sp., *Cupriavidus*, some members of the phylum Gemmatimonadetes (*Gemm 1*, *Ellin5301*), and *Paenibacillus* were selectively depleted in nature farming soil and enriched in conventional farming soil (Figure 4A). Furthermore, some beneficial taxa, such as nitrogen-fixing *Rhizobium* and *Mesorhizobium*, *Streptomyces*, and *Burkholderia*, were highly associated with non-fumigated soils as keystone taxa (Figure 4C), while *Herbaspirillum*, some members of *Shingobacteriaceae*, *Amycolatopsis*, and *Bosea* were keystone taxa in fumigated soils (Figure 4C). Additionally, the degree of taxa enrichment varied depending on the cultivar involved. For example, ASV_002 (*Bacillus flexus*) was consistently enriched in conventional soil but was more abundant in mutants grown in fumigated conventional soil (Supplementary Figures 4A, 5; Supplementary Table 5), while ASV_020 and ASV_021 (*Burkholderia bryophila* and *Sphingomonas* sp., respectively), were consistently enriched in the rhizosphere of wild-type and mutants, grown in non-fumigated nature farming soils, respectively (Supplementary Figures 5A, 6; Supplementary Table 5).

As for the fungal community, keystone taxa in the rhizosphere of plants grown in nature farming soil included *Metarhizium* sp. (Figure 4B), which was also differentially abundant in non-fumigated soils (Figure 4D). Conversely, *Neurospora* was significant in nature and fumigated soils. *Mortierella* sp. was also among the taxa discriminatively recruited in the rhizosphere of both wild-type and mutants grown in non-fumigated conventional farming soil (Supplementary Figures 5B, 7; Supplementary Table 6). On the other hand, fungal ASV_039 (*Chaetomium grande*) was discriminatively higher in plants associated with fumigated conventional soil but more abundant in the rhizosphere of wild-type soybean (Supplementary Figures 5B, 7; Supplementary Table 6).

### 3.10. Functional prediction analysis of soybean rhizosphere bacterial communities

**Figure 5:**
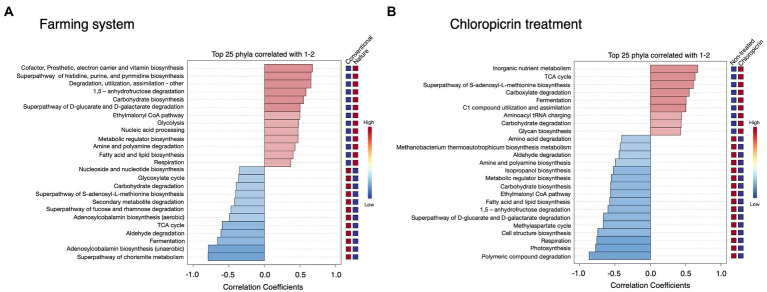
Putative functional prediction of bacterial communities in the rhizosphere of soybean grown under different farming systems **(A)**, and the treatment of chloropicrin **(B)** using PICRUSt. The top 25 significantly dominant functional pathways are shown.

Phylogenetic Investigation of Communities by Reconstruction of Unobserved States 2 (PICRUSt2) was used to predict the putative functional differences of the bacterial communities colonizing the soybean rhizosphere under both conventional and nature farming systems and with or without fumigant treatment. Metabolic pathways related to cofactor and vitamin biosynthesis, and pathways related to degradation, utilization, and assimilation of compounds as sources of nutrients and energy, including amine and polyamine degradation, fatty acid and lipid biosynthesis, and superpathway of D-glucarate and D-galactarate were predominantly associated with the rhizobacteria in nature farming soil (Figure 5A). This is important since the microbes use the nutrient and energy for their growth and proliferation within the rhizosphere of the plants in nature farming soil. Pathways such as Nucleoside and nucleotide biosynthesis, glyoxylate cycle, secondary metabolite degradation, and TCA cycle were more dominant in treatments related to conventional farming (Figure 5A). There were more predicted functions in the non-fumigated soil compared to the fumigated soil. For example, amino acid degradation, aldehyde degradation, amine, and polyamine biosynthesis, fatty acid, and lipid biosynthesis, and photosynthesis were more pronounced in the non-fumigated soils (Figure 5B). Pathways such as inorganic nutrient metabolism, carboxylate degradation, carbohydrate degradation, and glycan biosynthesis were more prominent in the fumigated soils (Figure 5B).

### 3.11. Co-occurrence network analysis of the soybean rhizosphere

**Figure 6:**
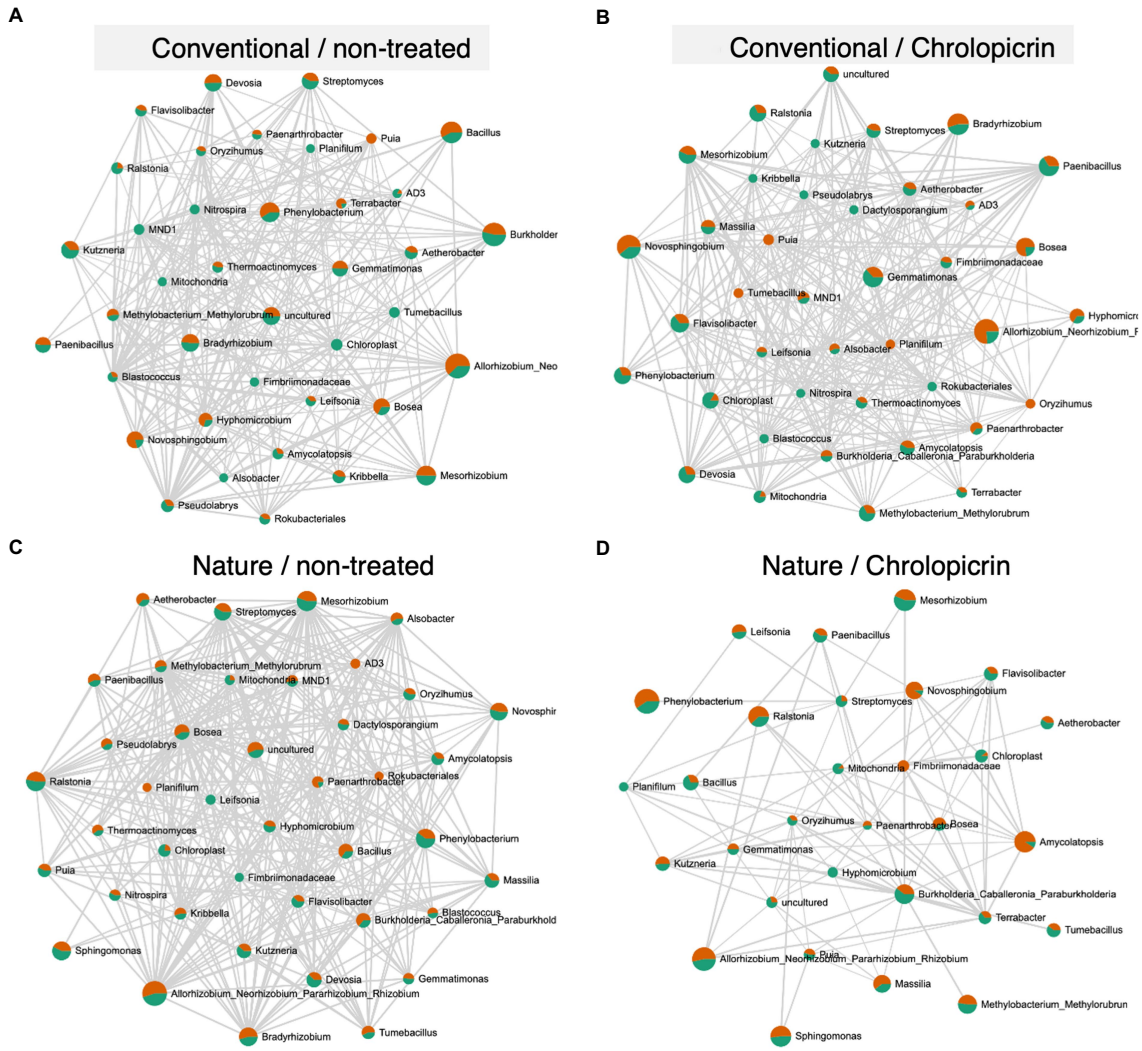
Network co-occurrence analysis of rhizosphere bacterial communities of soybean grown in non-treated conventional soil **(A)**, chloropicrin-treated conventional soil **(B)**, non-treated nature farming soil **(C)**, and chloropicrin-treated nature farming soil **(D)**. Correlation network generated using SparCC algorithm, with nodes representing taxa at the genus level and edges representing correlations between bacterial taxa. Co-occurrence networks are based on Pearson correlation with a threshold of *r* > 0.5.

The microbial communities in the non-fumigated nature farming soil were the most complex and densely connected than the respective networks of the conventional farming soil treatments, while the rhizosphere network was less connected and less dense in the fumigated nature farming soil (Figure 6). Positive pairwise interactions were observed in treated conventional farm soil between *Nitrospira* (belonging to phylum *Nitrospirota*) and *Mesorhizobium* but were negatively associated with *Rhizobium* and *Bradyrhizobium*, all of which belong to the phylum Proteobacteria (Figure 6B). *Rhizobium*, *Bosea*, *Mesorhizobium*, *Methylobacterium*, and *Bradyrhizobium* were among the most highly connected taxa co-occurring under non-fumigated nature farming soil (Figure 6C) and were all involved in positive interactions among themselves and with other taxa. The positive interactions with these taxa imply their synergistic activities in enhancing N_2_-fixation.

## 4. Discussion

Although the interaction of plant-microbe and microbe-microbe in the rhizosphere is well-studied in several crops (Edwards et al., 2015; Durán et al., 2018; Thiergart et al., 2019), the interaction of microorganisms in the rhizosphere of crops under nature farming systems is poorly understood. In the present study, the rhizosphere microbiome of soybean and its impact on soybean growth in nature farming soil was compared to conventional farming practices under soil fumigation treatment. The present study examined the rhizosphere microbiome to understand the impact of agrochemical inputs on beneficial soil microbes and plant growth, and how alternate farming practices like nature farming where no external fertilizers and pesticides are used can influence the abundance of beneficial soil microbes and growth of plants.

### 4.1. Impact of farming system and fumigation on symbiotic associations of soybean

In the present study, a significantly higher number of nodules was observed on the roots of wild-type soybean in the nature farming soil than in the conventional farming soil when no fumigant was applied (Figure 1A). This could be due to the lower indigenous Rhizobium populations in the conventional than the nature farming soil or the lack of soybean symbiotic compatible rhizobia strains in the conventional farming soil. A highly significant interaction was observed between farming system, fumigation treatment, and soybean cultivar on the number of nodules formed. This may imply the effect of the farming system or fumigant applied nodule formation is dependent on the soybean cultivar used as evident from nodules formed in the wild-type plants but not the mutant plants.

Arbuscular mycorrhiza fungi, the most common and highly beneficial plant-associated fungi (Smith et al., 2011), are of particular interest in nature farming systems, where soil fertility is maintained without the use of agrochemicals. Interestingly, a significantly higher percent root colonization in terms of hyphae, vesicles, and arbuscles was observed in non-fumigated nature farming treatments than in non-fumigated conventional farming treatments (Figures 1C,D). This is in line with other reports stating that plants grown in organically managed soils had higher AMF colonization rate in comparison to plants in high-input conventional farming soils (Entz et al., 2004; Gosling et al., 2010; Gazdag et al., 2019). This may be partially explained by the fact that AMF thrives in environments with low nutrient input as in nature farming where nutrients are slowly recycled by soil microorganisms. This is in contrast to conventional farming practices where large amounts of chemicals are frequently applied thereby impacting the soybean-fungal symbiotic interactions with respect to reciprocal exchange of nutrient and carbon between the two (Oehl et al., 2004). It should be noted, however, that the available P content was higher in the nature farming soil than in the conventional farming soil in our study (Supplementary Table 1). Thus, factors other than P, such as the population of viable AMF spores might have influenced AMF colonization in this study. Therefore, further studies are required to characterize the AM fungi species diversity and spore abundance in conventional versus nature farming practices before stronger conclusions can be made.

Fumigation with chloropicrin, on the other hand, significantly reduced AMF colonization and the number of root nodules, with a stronger effect on plants grown in nature farming soil (Figures 1A–C). This suggests that an active indigenous population of rhizobia and AM fungi existed in the nature farming soil, which was severely impacted by fumigation. From this results, it can be hypothesize that the fumigant negatively affected nodulation and consequently biological nitrogen fixation in the soybean by disrupting the signaling activities of *Rhizobium* needed for root infection or by affecting the root hairs of the soybean where infection and node formation occur (Meena et al., 2020). Previous research has shown that soil fumigants, such as chloropicrin and formaldehyde, inhibit AMF root infection and spore development (Dangi et al., 2017), which is attributed to the toxic effects of fumigants on the fungal spore and hyphal development. Even though there was no significant interaction between all the three variables involved in this study, there was a significant interaction (*p* < 0.001; Supplementary Table 2) between farming system and fumigation treatment on AMF colonization rates. Thus, the effect on the fumigant on the AM fungi colonization was more dependent on the type of farming system, which could explain why the fumigant had a more severe impact on AM fungi colonization in nature farming irrespective of the soybean cultivar. This means there could be different species of AM fungi present in either of the two farmed soils that are either more susceptible or tolerant to the fumigant due to the farming management practices undertaken in conventional or nature farming.

### 4.2. Farming system and fumigation dependent growth performance of soybean

In this study, the plant heights of wild-type soybean growing in conventional, or nature farming soil were not significantly different irrespective of fumigation treatment but were all significantly higher than the mutant plant treatments (Table 1). Since all soil systems were the same for both cultivars, this effect could be attributed to the genetic makeup of the mutant that resulted in restricted nodule formation and rhizobial symbiosis. It was shown in this study that the soybean root weight was relatively not impacted by either the farming system or fumigant application except in mutant plants in the non-fumigated conventional farming soil (Table 1). This could be attributed to the eradication of root pathogens by the fumigant around the roots of the soybean, or the presence of biocontrol microbes in the nature farming soil, which reflects how the crop should look in a healthy soil system.

Leaf chlorophyll content in the mutant increased in the fumigated soils (conventional and nature) compared to those in the non-fumigated soil and was similar to wild-type plants (Table 1). In the mutants grown in the non-fumigated soils, lower leaf chlorophyll content is not surprising because of the lack of nitrogen fixation as a result of the absence of nodule formation (Figures 1A,B). However, previous studies have found that fumigation increased the nitrogen content of soils because of the degradation and release of nutrients from the dead microbial cells, which may explain the higher chlorophyll content in mutant plants associated with the fumigated soils (Yan et al., 2022). Furthermore, the end products of chloropicrin, which was the fumigant used in this study, upon degradation are carbon dioxide, chlorine, and nitrogen. Therefore, the release of nitrogen after the degradation of chloropicrin may also explain the increased leaf chlorophyll content of mutant plants grown in fumigated soils in comparison to mutants in non-fumigated soils. The current study was however limited in soil nutrient analysis of the fumigated soils and plant nutrient uptake after harvest which should be considered in future studies. Interestingly, we observed a more pronounced and significantly higher average pod and seed number in the soybean plants grown in the nature farming soil in the mutant, but the number decreased significantly in the fumigated treatment (Table 1). It can therefore be postulated that this effect might be a result of the presence of some beneficial microbial symbionts such as AMF within the mutant’s rhizosphere that helped in promoting the pod and seed formation relative to the wild-type plants because there was no rhizobia symbiosis for nodule formation in the mutant.

### 4.3. Farming management practices shape the soybean rhizosphere community

While the crop variety had a smaller impact on the soybean rhizosphere microbiome diversity, farming practices had a major influence on the same. Therefore, most discussions below were focused on the farming system. The current study demonstrated that the rhizosphere of the naturally managed soybean plants had greater bacterial and fungal diversity (Shannon and Chao1) than the conventionally managed soybean (Figures 2A,B; Supplementary Tables 3, 4). Similar to this study, others have found a higher microbial diversity in organic/nature farming systems compared to conventional systems (Chaudhry et al., 2012; Hartmann et al., 2015; Lupatini et al., 2017; Ares et al., 2021; Sinong et al., 2021). Higher alpha diversity in the nature farming rhizosphere soil is also found in soil where there is restricted use of agrochemicals. Lupatini et al. (2017) reported that the continuous use of agrochemicals under conventional farming practices can directly or indirectly eliminate and inhibit the growth of certain microbial groups resulting in lower microbial diversity in conventional soils compared to organically managed soils.

Interestingly, the alpha diversity analyses showed that, the mutant’s fungal diversity was significantly higher in the conventional farming, non-fumigated soil than in the nature farming soil (Figure 2B; Supplementary Table 4). It is possible that there is a selective effect of root exudates secreted by the mutant plants for specific taxa that may be beneficial for their growth and health as has been observed before (Mendes et al., 2013; Philippot et al., 2013). There was a distinct separation of the rhizosphere bacterial and fungal community between the conventionally managed and naturally managed soil while the effect of soybean cultivar was negligible (Figures 2C,D; Supplementary Figure 3). Although plant cultivar did not have a strong effect on the structure of the rhizosphere communities in the current study, studies in other systems have identified such an effect (Ares et al., 2021). The results from this study showed that, independent of plant cultivar, farm management practices can have a marked impact on the rhizosphere microbiome structure of plants.

### 4.4. Soil fumigation deleteriously affects microbial diversity and alters the soybean rhizosphere community structure

Some studies have reported that soil microbiomes are considerably altered by chemical fumigants (Ibekwe et al., 2001; Rokunuzzaman et al., 2016; Li et al., 2017). In this study, it was observed that the applied soil fumigant significantly reduced the soybean rhizosphere microbial diversity compared to non-fumigated treatments under both farming systems (Figures 2A,B; Supplementary Tables 3, 4) possibly because the toxicity of the fumigant led to increased cell mortality, thus preventing the recovery of some microbial taxa and resulting in a considerable decline in microbial richness and diversity (Xiong et al., 2022). Several other studies have reported lower microbial alpha diversity because of soil fumigation and attributed it to the direct toxicity of fumigants, which have broad-spectrum biocidal activity against some indigenous soil microorganisms (Ibekwe et al., 2001; Rokunuzzaman et al., 2016; Li et al., 2017).

This study demonstrated that the rhizosphere communities in the conventional farming soil treated with fumigant had similar bacterial and fungal community structure with those in the fumigated nature farming soil. In nature farming soil, however, the rhizosphere communities in fumigated samples differed from those in non-fumigated samples for bacteria and fungi (Figures 2C,D). This is most likely because the rhizosphere community in the conventional soil was more resistant to the stress from the fumigant possibly because of an adaptation mechanism developed from the continuous use of chemicals under conventional farming practices. In contrast, because various microbes have varying degrees of tolerance to external stressors (Bouskill et al., 2013), exposing the nature farming soil to fumigation caused a shift in the rhizosphere microbial profiles toward a more resistant taxa that could endure the fumigant stress and occupy the empty niches, resulting in two distinct communities (Figure 2C,D; Supplementary Figure 3).

### 4.5. Influence of farming management practice on the taxonomic composition of soybean rhizosphere

Proteobacteria was the most predominant bacterial phylum (ranging from 34.5 to 65.8%) found across all the rhizosphere soil samples in our study (Supplementary Figure 4A). Members of Proteobacteria are generally considered as copiotrophs that are adapted to C-rich environments for their fast growth and propagation (Ling et al., 2022). A trait of the rhizosphere similar across diverse plant species is the secretion of root exudates that provide a root-derived carbon substrate for rhizospheric microorganisms. It is therefore not surprising that the phylum Proteobacteria was consistently enriched even in the fumigated rhizosphere samples. Firmicutes, although present in the rhizosphere of plants of conventional and nature farming soils, was considerably enriched in the conventional than the nature farming soil (Supplementary Figure 4A). Members of this phylum, including *Bacillus* are known to produce antibiotics that control a wide range of soil-borne plant pathogens. Acidobacteria which were more enriched in the rhizosphere of naturally managed plants, are generally considered as oligotrophs that are dominant in soils with low resource availability (Fierer et al., 2012). This may explain their enrichment in the rhizosphere soil of the naturally managed soybean plants.

*Rhizobium*, *Phenylobacterium*, and *Metarhizium* in the nature farming soil and *Bacillus*, *Cupriavidus*, *Mortierella*, *Chaetomium*, and *Curvularia* in the conventional farming soil were among the bacterial and fungal taxa that contributed to differentiate the soybean rhizosphere communities associated with the two farming systems according to the LEfSe analysis (Figures 4A,B). Beyond their contributions to legume nodulation and nitrogen fixation, some rhizobium species have been reported to promote plant growth aiding in P solubilization (Ahmad et al., 2008) and production of indole-acetic acid (IAA), which promotes root elongation for nutrient absorption (Igiehon et al., 2019). Quite recently, Baron et al. (2020) reported the ability of *Metarhizium* species *Metarhizium marquandii* to produce IAA and solubilize insoluble phosphate while also promoting the growth of soybean.

### 4.6. Influence of soil fumigation on the taxonomic composition of soybean rhizosphere

The results showed that fumigation led to changes in the soybean rhizosphere microbiome composition by increasing or decreasing the relative abundances of certain taxa compared to the non-fumigated samples. In the bacterial communities, the relative abundance Proteobacteria decreased slightly in abundance across all the rhizosphere samples while Acidobacteria was inhibited in the nature farming rhizosphere samples after fumigation (Supplementary Figure 4A). Previous studies reported that members of the phylum Acidobacteria are often sensitive to environmental stressors (Xiong et al., 2022), and this sensitivity could be a possible reason for their inhibition in the rhizosphere soils after fumigation. The relative abundance of Firmicutes increased after fumigation with higher proportions in the conventional farming rhizosphere samples than the nature farming samples (Supplementary Figure 4A). Liu et al. (2015) also found increased proportions of gram (+) bacteria in the soil after fumigation and reported that these microbial groups are more tolerant to chemicals including fumigants because of their cell wall composition and ability to form spores, which make them resist external stressors and enable them to adapt more quickly to vapor from fumigants.

The bacterial genera *Rhizobium*, *Burkholderia*, and *Streptomyces* were the specific taxa that contributed the most toward a significant differentiation of the rhizosphere microbial communities after fumigation and the fungal genera *Trichoderma*, *Clonostachys*, *Mortierella*, and *Metarhizium* toward the non-fumigated samples (Figures 4C,D). *Burkholderia bryophila* which was discriminately enriched in nature farming soil, has been reported to exhibit antagonistic activity against several plant pathogens including *Xanthomonas campestris* and *Rhizoctonia solani* promotes the production of plant growth-promoting hormones such as ACC deaminase and siderophore (Vandamme et al., 2007). *Streptomyces* is known for the biocontrol of soilborne fungal pathogens because of its antagonistic activity through the synthesis of various antifungal metabolites (Viaene et al., 2016; Vurukonda et al., 2018) and its role in inorganic phosphate solubilization (de Jesus Sousa and Olivares, 2016). Trichoderma is a plant-growth-promoting fungus that enhances plant nutrient uptake, produces growth hormones, and protects plants from pathogen infection (Bononi et al., 2020; Chen et al., 2021). The genus *Mortierella* is known to produce antibiotics and acts as a potential antagonist against various plant pathogens (Xiong et al., 2017). Fumigation however contributed to a significant decrease in the abundance of these beneficial microorganisms which further confirms the impact of chemical fumigants on eliminating or inhibiting non-target beneficial microorganisms in the soil.

The LEfSe analysis revealed that the bacterial genera *Herbaspirillum*, and *Bacillus*, and the fungal genera *Neurospora* and *Chaetomium* responded positively to the fumigant applied and were significantly enriched in the soybean rhizosphere samples (Figures 4C,D). The increased abundance of these microbial taxa after fumigation may be linked to their ability to degrade and utilize the fumigant as a nutrient source for their growth and for colonizing empty niches (Castellano-Hinojosa et al., 2021). A *Neurospora* sp. with phosphate solubilizing ability was isolated from the rhizosphere of *Sorghum bicolor* L. (Sharma et al., 2016). Outlining the exact functions of *Neurospora* sp. in the soybean microbiome would require further experimentation and functional characterization. *Chaetomium*, another fungus that was significantly enriched in the fumigated treatments, has been widely used as a biocontrol agent (Castellano-Hinojosa et al., 2021), suggesting that the soybean plants recruited these fumigant-tolerant organisms for their growth.

### 4.7. Putative functional traits of soybean microbiome predicted by PICRUST2

The functional prediction analysis in this study revealed that the rhizosphere microbiome of plants in the naturally managed soil can be associated with more putative functions than in conventionally managed soil (Figure 5A), which may be related to a higher microbial diversity observed in the rhizosphere of plants in the naturally managed soil than those in the conventionally managed soil. Furthermore, more diverse metabolic functions were associated with the rhizosphere microbiome of plants in non-fumigated soils than in fumigated soils (Figure 5B). This is understandable because we observed higher rhizosphere microbial diversity in plants associated with non-fumigated soils as compared to plants in fumigated soils, thus more diverse microbial communities may be responsible for carrying out more diverse functions. However, further analysis such as transcriptomics would give an improved understanding of the microbial communities and their functional activities.

### 4.8. Microbial co-occurrence network patterns of the soybean rhizosphere

Network analyses was performed to gain a more understanding of the complexity of microbial interactions operating within the various rhizosphere soil samples. The microbial networks in the nature farming rhizosphere soil with no fumigant was denser and had a more complex network with higher number of nodes and edges (Figure 6C) than those of the conventional farming treatments or the fumigated nature farming soil. This is undoubtedly related to the higher microbial diversity in the rhizosphere of naturally managed soybean plants. The results clearly show that farming practices have an impact on the complexity of the rhizosphere microbiome and that nature farming promotes more intricate interactions among different taxa, thus providing higher community stability. Our findings are similar to that of Banerjee et al. (2019) who observed significantly higher connected microbial communities in organically farmed soil than in conventional and no-till farming soil. Complex networks with high connectivity are more resilient to environmental stressors than simple networks with limited interactions (Banerjee et al., 2019). Thus, the higher complexity of rhizosphere microbial network structure in the nature farm soil may suggest that the different taxa in the rhizosphere of plants in nature farming soil may complement each other to withstand environmental perturbations such as abiotic stress or pathogen attack. The results further demonstrated that nature farming harbored a unique microbiome that was significantly impacted by the fumigant compared to the conventional farming soil microbiome. However, this finding needs further verification from analysis of a wider variety of nature farming soils from different farm locations and functional analysis of the obtained microbiota (to see how it is related to nutrient absorption and disease resistance) to make a valid conclusion on this hypothesis.

## 5. Conclusion

The present study investigated soybean growth and rhizosphere microbiome from conventional and nature farming soils, as well as the effect of soil fumigant on rhizosphere microbial communities. This is the first study that reports the effect of soybean rhizosphere microbial communities on plant growth under the nature farming system. The results revealed that in comparison to conventional farming soil, there were highly diverse bacterial and fungal communities in the rhizosphere of plants grown in nature farming, promoting a strong symbiosis of rhizobia and AMF. These are the most important bacteria and fungi microsymbionts that aid in nutrient acquisition and plant resilience even without the use of chemical fertilizers and pesticides. The results from this study confirmed a higher network complexity and node connectivity in nature farming soil without chloropicrin treatment, which may be indicative of a more robust community structure resistant to environmental stressors. Furthermore, we demonstrated that soil fumigant altered the rhizosphere microbiome’s composition developed around the soybean roots by eliminating or reducing the abundance of some beneficial plant-associated microbes including *Rhizobium*, *Streptomyces*, *Burkholderia* compared to the non-fumigated soil. The present study expands our knowledge of the uniqueness of nature farming and provides new insights for our understanding of positive microbial interactions within the rhizosphere of plants cultivated under nature farming practices.

## Data availability statement

The datasets presented in this study can be found in online repositories. The names of the repository/repositories and accession number(s) can be found in the article/Supplementary material.

## Author contributions

KD: conceptualization, methodology, investigation, formal analysis, software, writing (original draft), and visualization. MY: conceptualization, methodology, writing (review and editing), and project administration. SO: conceptualization, resources, and writing (review and editing), supervision, project administration, and funding acquisition. All authors contributed to the article and approved the submitted version.

## Funding

This study was supported by the JSPS Kakenhi (19H02860 and 19 K22303). This paper is based on results obtained from the project JPNP18016 commissioned by the New Energy and Industrial Technology Development Organization (NEDO).

## Conflict of interest

The authors declare that the research was conducted in the absence of any commercial or financial relationships that could be construed as a potential conflict of interest.

## Publisher’s note

All claims expressed in this article are solely those of the authors and do not necessarily represent those of their affiliated organizations, or those of the publisher, the editors and the reviewers. Any product that may be evaluated in this article, or claim that may be made by its manufacturer, is not guaranteed or endorsed by the publisher.
