# Supplementary material for: Diversity and function of soybean rhizosphere microbiome under nature farming
Source: Front Microbiol. 2023 Mar 1;14:1130969. doi: 10.3389/fmicb.2023.1130969 (PMC10014912; doi:10.3389/fmicb.2023.1130969)
Supplement: Supplementary file 1 [file Table_1.docx]

Supplementary Table 1. The chemical properties of soils used in the present study

EC = Electrical conductivity, TC = Total carbon, TN = Total nitrogen, N-NH_4_^+^ = Ammonium nitrogen, N-NO_3_^-^ = Nitrate nitrogen, C/N = Carbon nitrogen ratio, Avail.P = Available phosphorus
