# Supplementary material for: Diversity and function of soybean rhizosphere microbiome under nature farming
Source: Front Microbiol. 2023 Mar 1;14:1130969. doi: 10.3389/fmicb.2023.1130969 (PMC10014912; doi:10.3389/fmicb.2023.1130969)
Supplement: Supplementary file 3 [file Table_3.docx]

Supplementary Table 3. Alpha diversity (Shannon and Chao1) of rhizosphere bacterial microbiome of soybean grown in conventional and nature farming soils with and without chemical fumigant.
