# Supplementary material for: Diversity and function of soybean rhizosphere microbiome under nature farming
Source: Front Microbiol. 2023 Mar 1;14:1130969. doi: 10.3389/fmicb.2023.1130969 (PMC10014912; doi:10.3389/fmicb.2023.1130969)
Supplement: Supplementary file 7 [file Data_Sheet_1.docx]

Supplementary Figure 1. Examples of photomicrograph of arbuscular mycorrhizal fungi structures used as indicators of soybean (Enrei. Cv, wild-type and En1282, non-nodulating mutant) root colonization in conventional and natural faming soil after staining of roots with trypan blue solution. (h) hyphae, (v) vesicle, and (a) arbuscles.


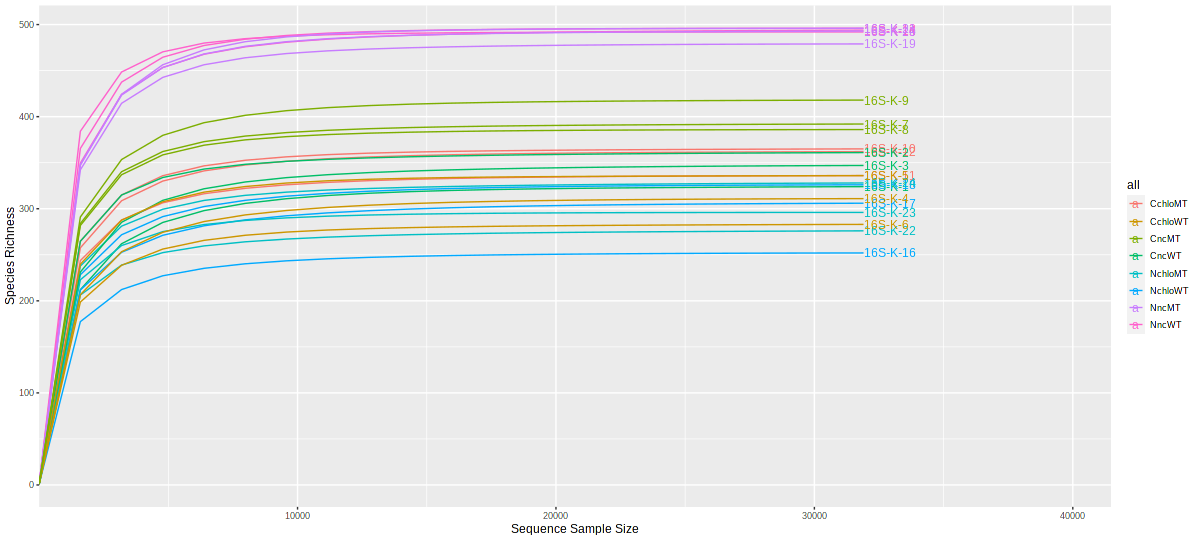


(A)


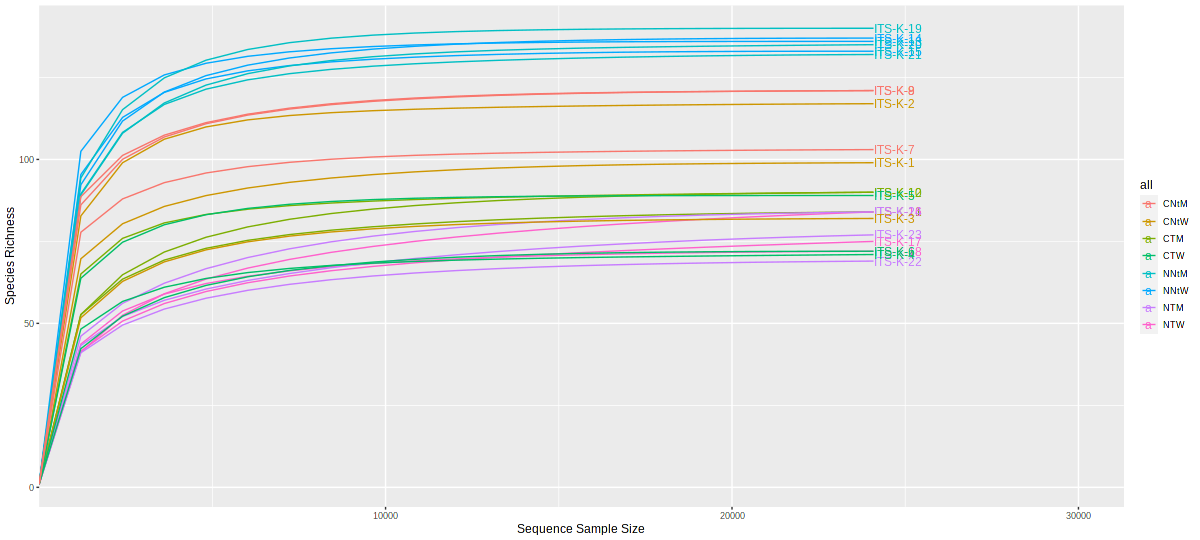


(B)

Supplementary Figure 2. Rarefaction curve of (A) Bacteria (B) Fungi

(B)

(A)

(C)


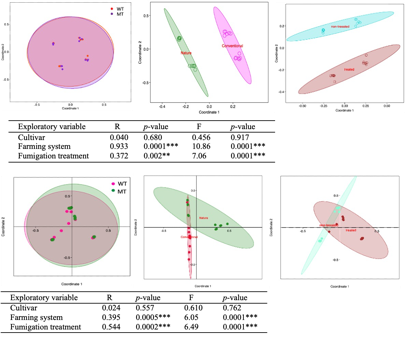


(D)

(G)

(F)

(E)

(H)

Supplementary Figure 3. PCoA, ANOSIM and PERMANOVA based analysis of the bacterial and fungal rhizosphere community structure as influenced by soybean cultivar (A, E), farming system (B, F), and fumigation treatment (C, G). ANOSIM and PERMANIOVA analysis of bacteria (D) and fungi (H).

(A)

(B)

Supplementary Figure 4. Taxonomic composition of microbial communities inhabiting the rhizosphere of soybean grown in conventional and nature farming soils with and without chloropicrin treatment. The relative abundance of (A) bacterial and (B) fungal communities at the phylum level. Only taxa with abundance >1% are shown. C = conventional farming soil, N = nature farming soil


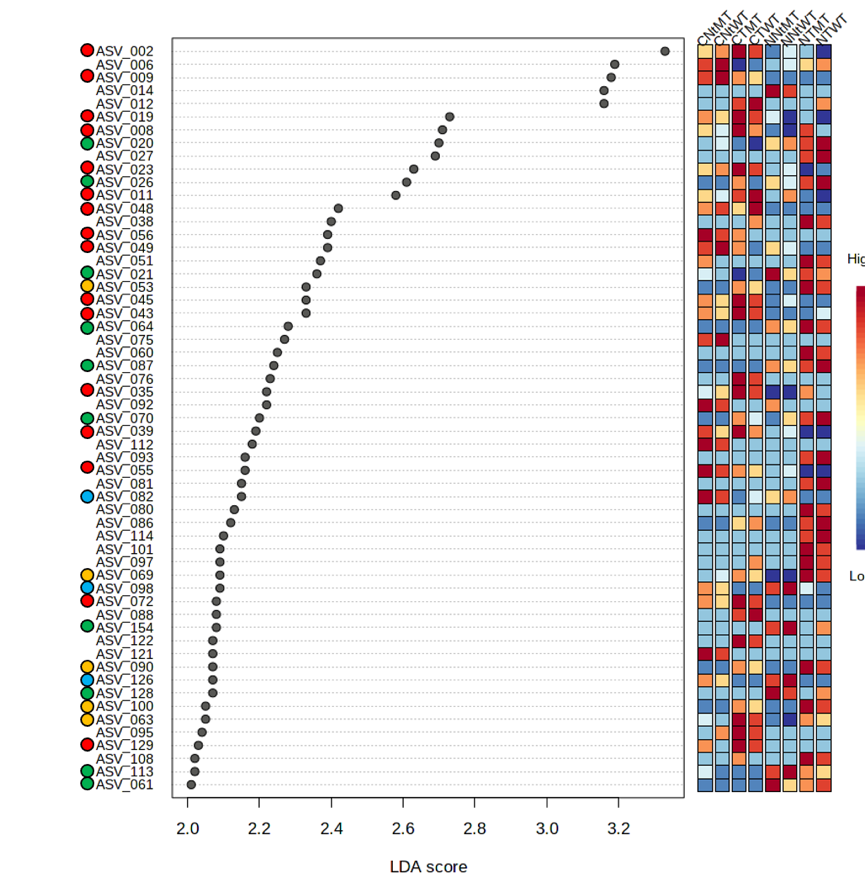


(A)


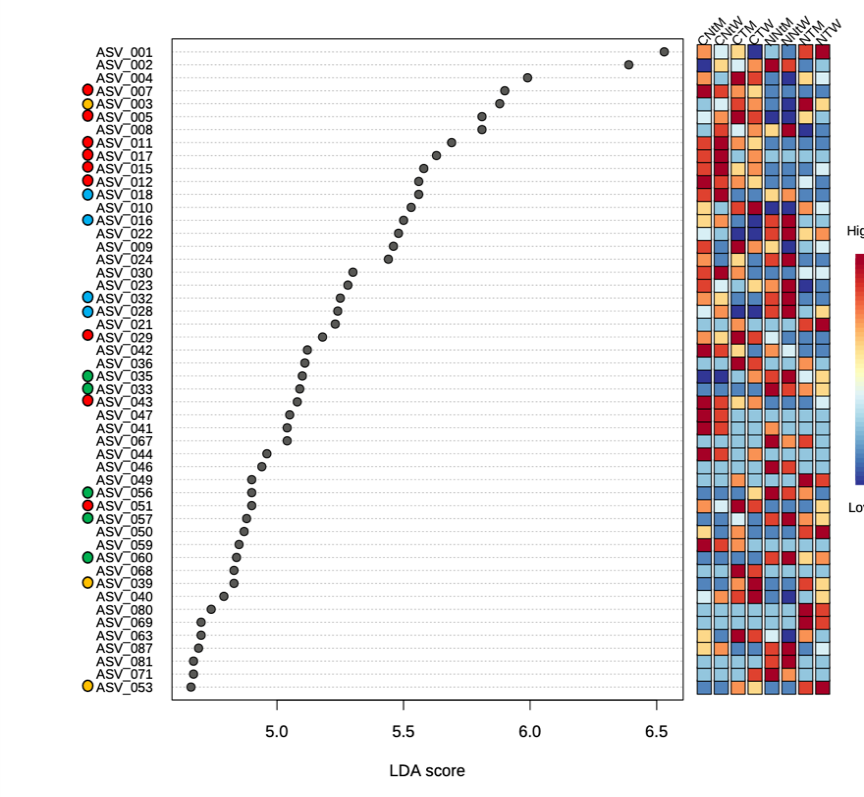


(B)

Supplementary Figure 5. Linear discriminant analysis (LDA) effect size (LEfSe) of (A) bacterial (B) fungal taxa significantly abundant in soybean rhizosphere as influenced by farming system and chloropicrin treatment. Colored circles besides the ASVs indicated farming system or chloropicrin treatment. Red = conventional farming soil, Green = nature farming soil, Blue = non treated soils, Yellow = fumigated soils. C = conventional farming soil, N = nature farming soil, Nt = non-treated, t = treated, WT = Enrei cv. (wild-type), MT = En1282 (non-nodulating mutant).


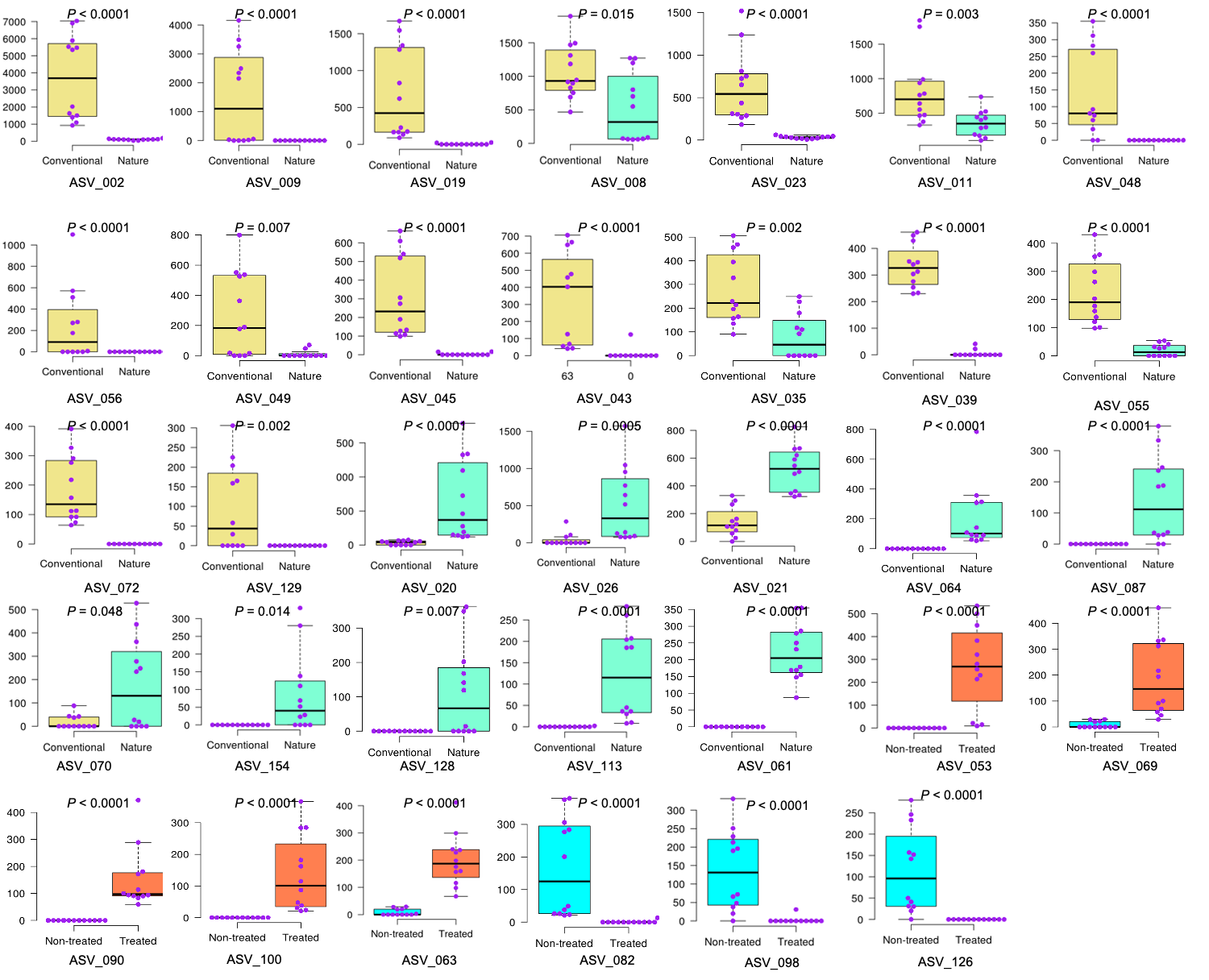


Supplementary Figure 6. Box plots representing significant bacterial taxa in the soybean rhizosphere as influenced by farming system and chloropicrin treatment


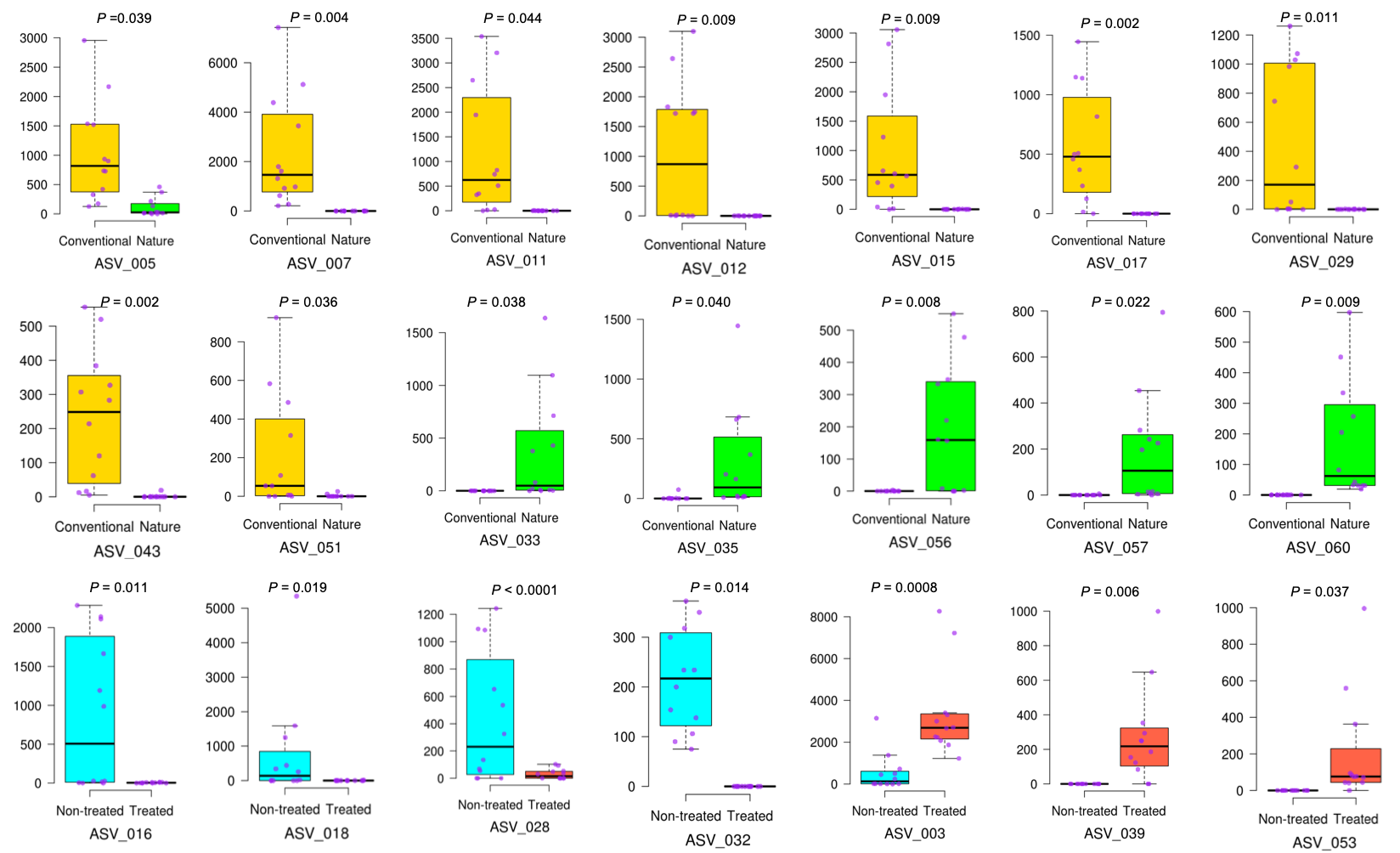


Supplementary Figure 7. Box plots representing significant fungal taxa in the soybean rhizosphere as influenced by farming system and chloropicrin treatment
